# A Fully Edible Transistor Based on a Toothpaste Pigment

**DOI:** 10.1002/advs.202404658

**Published:** 2024-09-16

**Authors:** Elena Feltri, Pierluigi Mondelli, Bojan Petrović, Fabrizio Mario Ferrarese, Alina Sharova, Goran Stojanović, Alessandro Luzio, Mario Caironi

**Affiliations:** ^1^ Center for Nano Science and Technology Istituto Italiano di Tecnologia Via Rubattino 81 Milano 20134 Italy; ^2^ Department of Physics Politecnico di Milano Piazza Leonardo da Vinci 32 Milano 20133 Italy; ^3^ Faculty of Medicine University of Novi Sad Hajduk Veljkova 3 Novi Sad 21000 Serbia; ^4^ Faculty of Technical Sciences University of Novi Sad T. Dositeja Obradovića 6 Novi Sad 21000 Serbia

**Keywords:** copper phthalocyanine, edible electronics, edible semiconductor, EGOFET, organic transistor

## Abstract

Edible electronics is emerging in recent years motivated by a diverse number of healthcare applications, where sensors can be safely ingested without the need for any medical supervision. However, the current lack of stable and well‐performing edible semiconductors needs to be addressed to reach technological maturity and allow the surge of a new generation of edible circuits. In the quest for good‐performing edible semiconductors, this study has explored the possibility of considering materials that are not regulated for intentional ingestion, yet are daily swallowed with no adverse reactions, such as pigments contained in toothpaste. This work first elaborates on the basis of inadvertent ingestion data to estimate the quantity of daily ingested Copper(II) Phthalocyanine (CuPc), a whitening pigment and well‐known organic semiconductor. Subsequently, CuPc is employed in the first demonstration of fully edible electrolyte‐gated transistors operating at low voltage (<1 V), displaying good reproducibility and stable performance for over a year. The results indicate that, with the daily ingested quantity of CuPc from toothpaste, more than 10^4^ edible transistors can be realized, thus paving the way to edible circuits, a critical component of future edible electronic systems.

## Graphical abstract

Edible electronics lacks reliable edible semiconductors, needed to enable active electronics components 100% safe for ingestion. Here, this study exploits Copper(II) Phthalocyanine, a pigment commonly found in toothpaste and consumed daily without adverse effects, and also recognized as a well‐established organic semiconductor, for the first demonstration of fully edible transistors operating at low voltage (<1V).

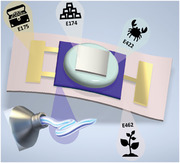

## 1 Introduction

As the workload of healthcare operators has increased in recent years, point‐of‐care gastrointestinal (GI) tract testing, i.e., the development of medical devices operating into the GI tract that do not need external supervision, could become increasingly crucial to ensure safe and early diagnosis, monitoring, and medical treatment. In this framework, inherently safe and edible devices can be key to overcoming limitations for non‐supervised administration posed by current ingestible technologies and allowing diffused and low‐impact point‐of‐care GI tract monitoring tools.^[^ ^1^, ^2^, ^3^, ^4^, ^5^, ^6^, ^7^, ^8^, ^9^, ^10^, ^11^, ^12^ ^]^

To date, a few electronic components and devices composed of food, food‐derived, and food‐like materials have already been proposed, including edible electrolytes and conductive pastes and inks,^[^ ^13^, ^14^, ^15^, ^16^ ^]^ honey‐gated circuits,^[^ ^17^ ^]^ defrosting sensors,^[^ ^18^ ^]^ edible batteries and supercapacitors,^[^ ^19^, ^20^, ^21^ ^]^ and edible sensors.^[^ ^22^, ^23^ ^]^ However, thus far, reliable and stable edible semiconductors, which are vital for the implementation of active microelectronic components in future edible systems, have not yet been identified. Although a large body of natural/synthetic dyes and pigments with suitable electronic structure (i.e., π‐conjugation and an optical bandgap in the visible range) is already being used in the food industry, the only food‐grade semiconductors qualified for charge transport in organic field‐effect transistor (OFET) architectures belong to the carotenoids family, like beta‐carotene (E160a^1^) and bixin (E160b).^[^ ^24^, ^25^ ^]^ Nevertheless, their limited performances and stability to ambient air and light exposure currently prevent them from being used in edible electronic applications.^[^ ^26^, ^27^ ^]^

To find edible materials with suitable semiconducting properties, we considered screening materials that are not used in the food industry but underwent toxicological tests on the ingestion route.^[^ ^28^ ^]^ This is the case for example for all cosmetics that, upon use, come into contact with mucous membranes at body openings, such as the mouth, or are inevitably ingested, such as toothpaste and lipsticks.^[^ ^29^, ^30^, ^31^, ^32^, ^33^, ^34^, ^35^, ^36^ ^]^ Among such materials, Copper(II) Phthalocyanine (CuPc) is one of the most popular commercial blue pigments, owing to its color brilliance, light fastness, stability, and low cost.^[^ ^37^, ^38^ ^]^ It is present in nail paints, soaps, and, most importantly, in toothpaste, where it is used as a whitening agent under its color index code C.I. 74160.^[^ ^39^, ^40^, ^41^, ^42^ ^]^ Commercial toothpaste formulations including CuPc have been on the market for more than 15 years, showing no reported side effects related to the pigment, making CuPc a robust candidate as a semiconductor in devices intended for ingestion.^[^ ^39^, ^40^, ^41^, ^43^, ^44^, ^45^, ^46^ ^]^

Due to the favorable sp^2^‐conjugated structure,^[^ ^47^ ^]^ molecular planarity, good air stability, and a demonstrated field‐effect charge carrier mobility up to 0.7 cm^2^ V^−1^ s^−1^ in thin films,^[^ ^48^, ^49^, ^50^ ^]^ CuPc gained great attention among small molecule semiconductors from the early days of organic electronics up to recent years.^[^ ^48^, ^51^, ^52^ ^]^ It has been widely employed as a p‐type semiconductor in electronic devices, such as organic solar cells^[^ ^51^ ^]^ and OFETs,^[^ ^48^, ^53^, ^54^ ^]^ with recent advancements extending to the field of biotechnology and biomedicine with biosensors for glucose determination and DNA sensing.^[^ ^53^, ^54^ ^]^

In this work, we propose the use of CuPc as the semiconductor for edible electronic devices. A twofold validation effort was undertaken on the one hand, aimed at supporting a non‐toxicity claim for the ingestion of edible electronic components incorporating CuPc as the active material, and on the other at demonstrating the feasibility of the fabrication of efficient CuPc‐based fully edible transistors. We first demonstrate, both through laboratory simulation tests and clinical data review, that no less than ≈0.5 mg of CuPc is ingested daily when using CuPc‐filled whitening toothpaste, which is orders of magnitudes more than the amount of semiconductor typically required to fabricate a single OFET.^[^ ^55^, ^56^ ^]^ To validate its use in the edible electronics field, we have then demonstrated that, upon structural fine‐tuning of CuPc thin films, a fully edible p‐type Electrolyte Gated OFET (EGOFET) can be realized. The device shows good electronic performances, with more than two orders of magnitudes of current modulation, stable operation in air over more than 1 year, and low‐voltage device driving (< 1 V), which is a fundamental aspect for the safe operability in in‐body applications, e.g. inside the GI tract. Overall, our approach sheds new light on a well‐known and stable organic semiconductor, allowing us to reconsider it in the context of edible electronics. Enriching the library of validated edible semiconductors is an important step to pave the way for integrating edible circuits in future edible electronic systems, such as intelligent food tags and point‐of‐care smart pills.

## 2 Ingestion Quantification of Copper(II) Phthalocyanine

Before entering the market, the toxicity of a chemical intended for cosmetics must be assessed.^[^ ^28^ ^]^ This is done by either in vivo tests on animal models, both in acute dose and repeated dose for oral exposure, inhalation, dermal exposure, and any other possible contact routes or, when possible, in vitro alternative tests. Cosmetic non‐toxicity in humans must be monitored, which is done by randomized clinical trials and post‐market surveillance.

In the EU for example, the latter is performed after commercialization by each single country, which is responsible for extensive surveillance of the cosmetic product, to monitor the eventual occurrence of serious undesirable effects in humans, upon both short and long‐term exposure (EU Regulation (EC) No 1223/2009).^[^ ^57^, ^58^ ^]^ With this regard, the data obtained with this surveillance provide a reliable source of information on the safety of a formulation intended for the general public.

Clinical trials instead can be conducted either by the product‐issuing company or by independent research laboratories to assess the effectiveness of a formulation before commercialization, or to study the adverse effect of specific components of the cosmetic products in a control group. For instance, an optimal amount of fluoride in commercial toothpaste has been established thanks to extensive clinical trials‐based studies on the correlation between accidental fluoride intake from toothpaste and the incurrence of dental fluorosis.^[^ ^59^, ^60^, ^61^ ^]^

As of today, studies conducted on toothpaste and mouthwashes have identified fluoride as the sole responsible for any potential pathology upon product swallowing above a certain threshold.^[^ ^42^, ^58^, ^59^, ^60^, ^61^ ^]^ A small number of other toothpaste components, like sodium lauryl sulfate, can mildly irritate the skin and oral mucosa, causing allergic cheilitis, but the majority of ingredients in commercial formulations have not shown any adverse reaction.^[^ ^42^, ^62^, ^63^, ^64^ ^]^

**Figure 1:**
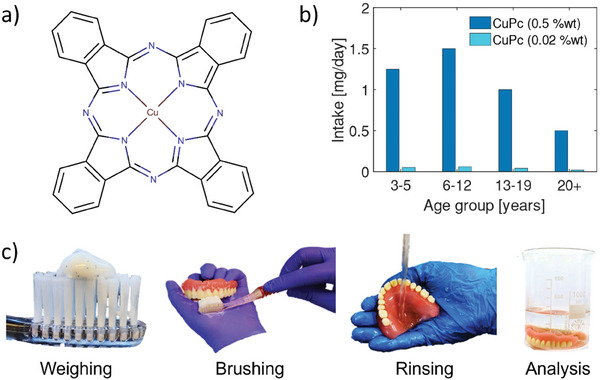
a) CuPc molecular structure. b) Histogram reviewing literature data on CuPc intake, expressed in mg per day, owing to accidental toothpaste ingestion, derived through patented CuPc concentrations (0.5% and 0.02% wt). c) Steps succession of the simulation experiment to quantify the amount of CuPc deposited onto the tooth enamel for whitening effect. 250 mg of CuPc‐rich toothpaste is placed on the toothbrush to brush a set of 28 real teeth. The teeth were brushed four times over two days, and, after being rinsed with deionized (DI) water, they were immersed in a 40 mL water solution. Lastly, via atomic absorption spectrometry, the amount of Cu inside the water solution is determined, which correlates with the amount of CuPc that is ultimately detached from the teeth enamel and consequently ingested.

Pigments fall in the latter class of not‐irritating components, and this is indeed the case for CuPc, an air‐stable pigment (reported in **Figure** **1**a) with assessed non‐toxicity (up to 6400 mg kg^−1^ bw) and low bioavailability.^[^ ^36^, ^65^, ^66^ ^]^ CuPc has been added in popular commercial toothpaste for more than a decade as whitening microbeads and neither clinical trials nor post‐market surveillance have ever flagged any reaction upon swallowing.^[^ ^39^, ^40^, ^41^, ^42^, ^43^, ^58^, ^67^ ^]^ Starting from a realistic estimation of the amount of swallowed toothpaste, the amount of CuPc safely ingested every day through toothpaste during a tooth brushing session can be roughly estimated.

To this aim, we have exploited the data reported in the book “Fluoride in Drinking Water: A Scientific Review of EPA's Standards”: in this work, the authors reviewed the scientific literature available on the topic of fluoride ingestion, with a section specifically dedicated to dental products.^[^ ^68^ ^]^ The authors analyzed and compared the results of more than 20 peer‐reviewed papers on toothpaste ingestion in the North American population from 1969 up until 2003, on more than a thousand subjects, the vast majority being children under the age of 10. The trial results on ingested fluoride are divided by age groups and used to estimate the amount of ingested toothpaste, considering the mean North American 1000 ppm fluoride content.^[^ ^60^ ^]^ Assuming the employed data set being current and generalizable, at least in countries with well‐established dental hygiene practices (out of socioeconomic factors) and to any formulation in the market, a rough, still meaningful, estimation of CuPc ingestion can be carried out (Figure 1b), knowing that the CuPc concentration in commercially available toothpaste ranges from 0.02 to 0.5% wt.^[^ ^39^, ^40^, ^41^ ^]^ We can ultimately state that: for the age group 3–5, between 0.05 and 1.25 mg of CuPc are ingested daily; for the 6–12 age group, between 0.06 and 1.5 mg; for the 13–19 age group, between 0.04 and 1 mg; while for adults over the age of 20, between 0.02 and 0.5 mg.

Nevertheless, a proper estimation of the pigment oral intake should not only account for the accidental toothpaste swallowing but also the pigment temporarily adhering to the teeth enamel, thus allowing the whitening action. Indeed, commercial whiteners made with optical pigments, such as CuPc, work on the principle that, by adding a thin, translucent layer to the enamel, the yellow discoloration is altered, making the teeth appear whiter and brighter thanks to blue being on the opposite side of the color spectrum to yellow.^[^ ^43^ ^]^ At the end of the tooth cleaning session, most of the toothpaste will be rinsed off and a small part will be swallowed. The CuPc whitening layer will instead stay anchored to the teeth to perform its whitening activity, until it gets eventually removed either mechanically or exfoliated by the saliva, and ultimately ingested.

To quantify the whitening layer covering the tooth enamel, we have developed an experimental method to simulate the brushing and rinsing stage on real human teeth. Figure 1c shows the main steps of the experiment: 250 mg of a commercial toothpaste, containing CuPc in the form of blue covarine microbeads, were placed on a toothbrush; a total of 28 human teeth, placed in two pairs of dental prostheses, were brushed in a non‐Arcon type semi‐adjustable articulator. The teeth were brushed four times over two days and, after being rinsed, were placed in DI water to collect the leftover whitening CuPc layer. The whole resulting solution was then analyzed via an atomic absorption spectrometer to determine the total amount of Cu. The obtained concentration of Cu in the solution was 2.5 mg kg^−1^. From this concentration value, knowing the molecular weight of Cu and CuPc, a total of 0.45 mg of CuPc is calculated to be ingested daily solely as a consequence of the whitening layer, which implies a mean CuPc amount of roughly 0.5% wt for the considered toothpaste.

In summary, we have evaluated the amount of ingested CuPc both from accidental toothpaste swallowing, by reviewing data from existing clinical trials, and from the intended thin whitening layer left on the teeth after rinsing, for which we have devised a targeted experiment. Our final estimation of the overall daily ingestion of CuPc is between ≈0.5 mg for adults using a toothpaste containing 0.02% wt of CuPc, to ≈2.0 mg for the case of children using a 0.5% wt CuPc containing toothpaste.

## 3 Optical and Morphological Characterization of Evaporated CuPc Films

Here we analyze the optical and microstructural properties of evaporated CuPc films to be subsequently used for the realization of edible transistors. CuPc film processing has been limited to thermal evaporation due to its insolubility in most organic solvents. There is a broad literature on the relation between the electrical performance of the resulting CuPc evaporated film, which ultimately depends on the film crystallinity and molecular arrangement, and the set of evaporation parameters used (i.e., evaporation rate, base pressure, substrate temperature).^[^ ^69^, ^70^, ^71^, ^72^, ^73^ ^]^ Low deposition rates (0.01–1 Å s^−1^), low base pressure (<10^−4^ mTorr), and high substrate temperatures (120–180 °C) are preferred conditions to obtain large crystallites and reduced grain boundaries, which represent the optimum condition for charge transport in common organic field‐effect transistors (OFETs).^[^ ^69^, ^71^, ^72^ ^]^ However, the substrate temperature window is quite limited in the framework of edible electronics, as it cannot be pushed above 100 °C due to the minimal temperature resilience of common edible substrates, generally relying on mechanically robust polysaccharides and derivatives.^[^ ^56^ ^]^ For this reason, we have adopted a rate of 0.05 Å s^−1^, and a base pressure between 3 × 10^−7^ and 6 × 10^−7^ mTorr for the CuPc evaporation on unheated substrates, in compliance with edible platforms.

**Figure 2:**
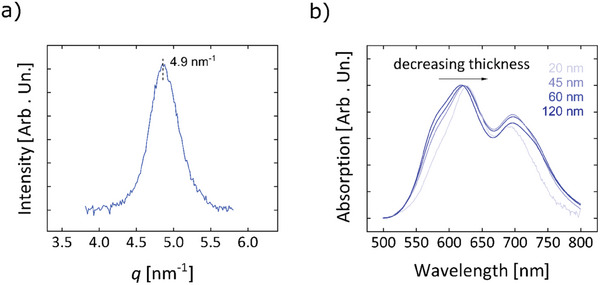
a) XRD diffractogram on a 60 nm‐thick CuPc film. b) UV–vis spectra evolution with the film thickness, considering thin films of 20, 45, 60, and 120 nm, suggesting a different CuPc aggregation in the first evaporated layers.

To unveil the predominant crystalline phase in our films, we have performed X‐ray Diffraction (XRD) experiments on CuPc films evaporated onto glass substrates. The main Bragg peak, centered at q ≈ 4.9 nm^−1^ (**Figure** **2**a; Figure S1, Supporting Information), is representative of the (2 0 0) reflection of the *α*‐phase. This indicates a preferential *edge‐on* molecular alignment with respect to the substrate, in agreement with previous observations on films grown at low substrate temperatures.^[^ ^74^ ^]^

Thickness‐dependent UV–vis absorption spectroscopy (Figure 2b) has revealed a slight wavelength redshift of the high‐energy peak of the Q band for thinner films. As the Q band is commonly attributed to the first *π*–*π* ^*^ transition on the phthalocyanine macrocycle,^[^ ^67^ ^]^ the redshift suggests a slightly different CuPc molecular packing at different thicknesses. A similar trend in the UV–vis has been observed in literature,^[^ ^75^ ^]^ where a brickstone CuPc *𝛼*‐phase was considered as the predominant arrangement in thick films (thickness > 10 nm), while the herringbone *𝛼*‐phase is representative of thinner films. We highlight that the herringbone packing is generally preferred over the brickstone as it exhibits more effective orbital overlap with a lower degree of anisotropy, thus favoring high‐mobility systems.^[^ ^76^ ^]^

## 4 Electrical Characterization of CuPc Evaporated Films

**Figure 3:**
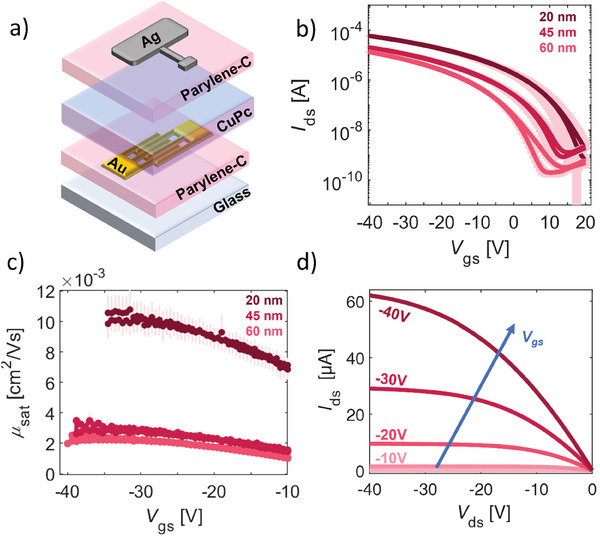
a) 3D sketch of the TGBC transistor with interdigitated inkjet‐printed gold contacts. with *W* = 27 mm and *L* = 20 µm on parylene‐C (450 nm). b) Mean *I‐V* transfer characteristics with standard deviation of the OFET devices with CuPc thin films of 20, 45, and 60 nm on seven devices per thickness, measured at *V*_ds_ = −40 V. c) Mean field‐effect mobility curves in saturation regime (*V*_ds_ = −40 V) with standard deviation for each CuPc thickness on seven devices per thickness. d) Output characteristics of a representative OFET device with a CuPc thickness of 20 nm with increasing applied gate voltage up to *V*_gs_ = −40 V.

To evaluate their charge transport properties, CuPc films with variable thicknesses, 20, 45, and 60 nm, were assessed at first in non‐edible top‐gate bottom‐contact (TGBC) reference OFETs, as sketched in **Figure** **3**a. Interdigitated gold source and drain contacts were inkjet‐printed on substrates made of electronic‐grade glass coated with a poly(chloro‐p‐xylene)‐C (parylene‐C) layer.^[^ ^56^ ^]^ The CuPc active layers were then deposited on the top of the source and drain contacts as described in Section 2. Parylene‐C coating was employed to promote the growth of CuPc crystallites and reduce grain boundaries impact on transport.^[^ ^48^, ^53^, ^69^, ^77^, ^78^, ^79^, ^80^, ^81^ ^]^ A parylene‐C layer (450 nm thick) also serves as the dielectric layer, on top of which an inkjet‐printed silver pattern was deposited as the gate electrode (see Figure S2, Supporting Information for a representative SEM cross‐sectional image). The fabricated devices have a channel length *L* of 20 µm and a *W* of 27 mm.

The mean current–voltage (*I*–*V*) transfer characteristic curves and the mean field‐effect mobilities curves versus gate voltage, *V*_gs_, measured at a fixed source‐drain voltage, *V*_ds_ = −40 V, of the CuPc reference devices with variable semiconductor thickness, averaged over seven devices per thickness, are reported in Figure 3b,c, respectively. All the devices demonstrated unipolar p‐type behavior, i.e., an increase in source‐drain current *I*_ds_ when decreasing *V*_gs_ (*V*_gs_ < 0 V). Irrespective of the CuPc thickness, OFETs present little to no hysteresis, typical of an ideal semiconductor/dielectric interface with negligible trap density.

We observed an increasing *I*_ds_, trend, with decreasing CuPc film thickness as reported in **Table** **1**: at *V*_gs_ = −40 V, from a mean value of 13 µA for the thicker devices to a mean value of 61 µA for the thinner devices. According to previous literature reports,^[^ ^76^ ^]^ the *I*_ds_ trend is likely correlated to the structural change associated with the CuPc thickness (see Section 2, Figure 2d), suggesting a more efficient charge transport of CuPc herringbone domains in thinner films with respect to the brickstone ones in the thicker films.^[^ ^76^ ^]^

**Table 1:** Experimental values of *I*_ds_, *V*_th,_ and *µs*_at_ for different CuPc film thicknesses (20, 45, and 60 nm).

	*I*_ds_ [µA]	*V*_th_ [V]	*µ*_sat_ [cm^2^ V^−1^ s^−1^] (at *V*_gs_ *– V*_th_)
CuPc 20 nm	61 ± 5.6	5.2 ± 2.5	1 × 10^−2^ ± 1.2 × 10^−3^
CuPc 45 nm	20 ± 1.3	0.8 ± 0.5	3.5 × 10^−3^ ± 3 × 10^−4^
CuPc 60 nm	13 ± 1.2	−3.4 ± 0.7	2.1 × 10^−3^ ± 2 × 10^−4^

Field‐effect mobility curves were computed in saturation regime (*V*_ds_ = −40 V): thickness‐dependent mobility values, *µs*_at_, were calculated, as shown in Table 1, with a maximum of 1 × 10^−2^ cm^2^ V^−1^ s^−1^ for the 20 nm CuPc films, reaching the lowest value of 2.1 × 10^−3^ cm^2^ V^−1^ s^−1^ for 60 nm‐thick films. As per the *I*_ds_, the consequent *µs*_at_ trend points toward a more efficient charge transport of the CuPc herringbone domain.^[^ ^76^ ^]^ The *µs*_at_ values were extracted at the maximum *V*_gs_ for which the devices can still be considered in saturation regime, taking thus into account the threshold voltage, *V*_th_, shift toward positive potentials while scaling down the CuPc thickness (*V*_th_ = 5.2, 0.8, and −3.4 V respectively for the 20, 45, and 60 nm films) (see Figure S3, Supporting Information for the corresponding *I*–*V* transfer characteristic curves accounting for the *V*_th_ shift). The field‐effect mobility curves are almost independent of the gate potential, denoting the quasi‐ideal behavior of the transistors, with no substantial dependency on the charge carrier density.^[^ ^82^, ^83^, ^84^, ^85^ ^]^ The *V*_th_ shift toward positive voltages when reducing the CuPc thickness, which renders the devices “normally on”, i.e., with net *I*_ds_ at *V*_gs_ = 0 V, could be ascribed to atmospheric oxygen doping.^[^ ^86^, ^87^, ^88^ ^]^ To further confirm the quasi‐ideality of the devices, in Figure S4 (Supporting Information), field‐effect mobility curves in the linear regime, calculated at *V*_ds_ = −5 V, are reported: the *µ*_lin_ values are indeed almost identical to those computed at *V*_ds_ = −40 V. The output characteristic curves reported in Figure 3d confirm the good field‐effect behavior, with the linear trend at low *V*_ds_ indicating ohmic charge injection between the printed gold contacts and the semiconductor.

Despite the substrate constraint imposed on our process (i.e., unheated substrate during the CuPc evaporation), the best‐extracted mobility of ≈10^−2^ cm^2^ V^−1^ s^−1^ for the 20 nm thick film well compares with the best reports on OFETs based on evaporated CuPc thin films on heated substrates.^[^ ^48^ ^]^ Remarkably, these mobilities are almost two orders of magnitudes higher than the best‐reported ones obtained on edible semiconductors, i.e., carotenoids, in non‐edible OFET configurations, which are ≈10^−4^ cm^2^ V^−1^ s^−1^.^[^ ^24^, ^25^ ^]^

## 5 Fully Edible Electrolyte‐Gated CuPc Transistors

In this section we report on the realization of EGOFETs made only of edible materials, exploiting the optimized CuPc films as the semiconductor. Metal electrodes made of inert silver and gold represent viable options for microelectrodes in edible electronics, owing to their regulation as food additives, as denoted by E numbers E174 and E175, and frequently employed in food decoration. In addition, to our knowledge, the edibility of parylene‐C, used as both substrate and dielectric layer has never been demonstrated, although it has been reported as biocompatible and safe upon ingestion.^[^ ^89^, ^90^ ^]^ Therefore we had to replace parylene‐C with a validated edible option. Recently, edible substrates have been demonstrated in the form of ethyl cellulose (EC) films,^[^ ^55^, ^56^ ^]^ which are easy‐to‐process, chemically, and mechanically robust cellulose derivatives, commonly used in the food industry (E462). For our purposes, the water insolubility of EC, coupled with its non‐porosity and low surface roughness (see Figure S5b, Supporting Information), marks it as the best candidate for the deposition of water based inks, such as our gold ink.

**Figure 4:**
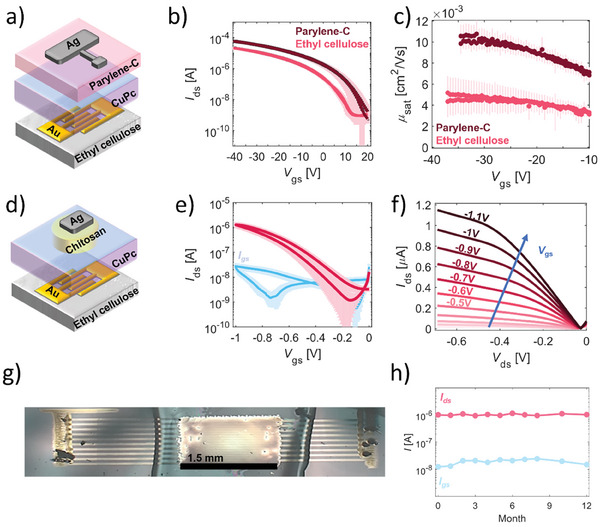
a) 2D sketch of an OFET architecture with EC as edible substrate. b) Comparison of *I‐V* transfer characteristics in saturation regime, *V*_ds_ = −40 V, of a 20 nm‐thick CuPc‐based OFET with parylene‐C and EC as substrate, averaged over seven devices each. c) Comparison of the corresponding field‐effect mobilities in saturation regime, *V*_ds_ = −40 V, over seven devices. d) 2D sketch of the fully edible OFET architecture with chitosan as edible electrolyte. e) *I*–*V* transfer characteristics with standard deviation in saturation regime (*V*_ds_ = −500 mV) of the chitosan‐gated EGOFET fully edible devices with a CuPc layer of 20 nm averaged over 20 devices. f) Output characteristic of a representative fully edible EGOFET. g) Photograph of a typical edible transistor. h) Shelf‐life stability over a year of a representative fully edible EGOFET monitored by recording *I*_ds_ and *I*_gs_ approximately once a month on a single transistor as a function of the passing months.

We first assessed the impact of the replacement of parylene‐C with EC at the interface with the semiconductor on the growth and the resulting transport properties of evaporated CuPc films. To this aim, we have fabricated OFET structures made with inkjet‐printed gold and silver metal contacts, EC as substrate, and parylene‐C as gate dielectric, with a 20 nm‐thick CuPc layer, as detailed in *Materials*. and sketched in **Figure** **4**a. Mean *I*–*V* transfer characteristic and corresponding field‐effect mobility in saturation regime averaged over seven OFETs are shown in Figure 4b,c. The field‐effect mobilities values were extracted at the maximum *V*_gs_ value for which the devices are still in saturation regime, taking into account the threshold voltage shift (*V*_th_ = 5.2 V for the devices with parylene‐C as substrate and *V*_th_ = 2.5 V for the ones having EC as substrate). An ideal and no hysteretic p‐type charge accumulation is shown in Figure 4b; however, a threefold *I*_ds_ drop for the EC‐based structure with respect to the film grown on parylene‐C substrates was observed. This reduction can be attributed to the altered surface energy of the substrate, as evidenced by contact angle measurements (Figure S6, Supporting Information), which indicates a more hydrophilic nature of the EC surface with respect to parylene‐C. The different wetting properties promote more elongated and well‐interconnected CuPc grains onto parylene‐C, as visible by Atomic Force Microscopy (AFM) in Figure S5 (Supporting Information).^[^ ^48^ ^]^ Nevertheless, we measured a current On/Off ratio higher than three orders of magnitudes, along with a mean mobility of 5.9 × 10^−3^ cm^2^ V^−1^ s^−1^, which well compares to the values obtained for CuPc films grown on non‐engineered and unheated substrates.^[^ ^48^, ^91^, ^92^ ^]^

Finally, we tackled the replacement of the dielectric with an edible one. The selection of the dielectric layer plays a key role in the development of application‐specific edible devices. For instance, in the context of creating point‐of‐care tools for monitoring activities in the GI tract, which hold great promise for edible electronic components, it is imperative to ensure efficient low‐voltage operation. This is essential to prevent any interference with the natural physiological functions of the GI system, thereby mitigating potential health risks.^[^ ^93^ ^]^ Therefore, a high capacitance dielectric layer is needed. High capacitance can be targeted by either: 1) using high‐k or ultra‐thin dielectrics, or, 2) using electrolytes exploiting the formation of nanometric electrical double‐layers (EDLs) at the interface between electrolyte and semiconductor.

Recently, Sharova et al. have demonstrated the feasibility of inkjet‐printed EGOFETs with an edible chitosan:glycerol‐based electrolyte for stable low‐voltage (< 1 V) operation.^[^ ^56^ ^]^ We tested the compatibility of such solution‐processed chitosan:glycerol electrolyte with our CuPc‐based devices in a fully edible structure, represented in Figure 4d,g. The *I*–*V* transfer characteristics of the resulting fully‐edible transistors are reported in Figure 4e: a p‐type current modulation is observed sweeping the gate current toward negative voltages, with *I*_ds_ maximum values of ≈1 µA and an On/Off ratio of ≈10^3^, averaged over 20 devices. We additionally performed electrochemical impedance spectroscopy (EIS) on a comparable stack, as detailed in Figure S7 (Supporting Information). By fitting EIS data, performed on the three different CuPc thicknesses and with four different DC biases, we estimated a capacitance value of 4 µF cm^−2^, which remained consistent across the three different CuPc film thicknesses (see Table S1, Supporting Information). Such evidence is in agreement with the formation of a conventional EDL at the interface between the semiconductor and the hydrogel, with no substantial permeation of ions in the semiconductor.^[^ ^94^, ^95^ ^]^ We then estimated the saturation field‐effect mobility to be ≈1.5 × 10^−3^ cm^2^ V^−1^ s^−1^, by imposing the capacitance to be the one extracted via EIS, and we calculated the transconductance normalized to the channel width W of the devices (g_m_/(W)) to be ≈10^−4^ S m^−1^, (see Figure S8, Supporting Information). Notably, the mobility falls within the same order of magnitude as the one measured in OFETs. *I*_ds_ drops by a factor of 2 with respect to the chitosan‐gated CuPc devices on top of parylene‐C substrates (reported in Figure S9, Supporting Information). This outcome is consistent with the difference observed in OFETs made on top of glass coated with parylene‐C and EC (as illustrated in Figure 4b), indicating that the adoption of chitosan:glycerol does not significantly compromise the transport characteristics of the semiconductor. In Figure 4f the output characteristic of the CuPc‐based fully‐edible EGOFETs is reported. Importantly, we observed that the fully edible devices offered a long‐term air shelf‐life of more than 1 year (Figure 4h) when stored in ambient conditions (T ≈25 °C and relative humidity between 40% and 50%), proving ambient air and light stability unparalleled by the edible semiconductors reported so far.^[^ ^27^ ^]^

In total, considering an active area of 1.5 mm^2^ and an optimized CuPc thickness of 20 nm along with a CuPc density of 1.6 g cm^−^^3^,^[^ ^96^ ^]^ each transistor carries 80 ng of semiconductor, which is well below the total amount of CuPc which is already ingested daily.

The other materials used in our devices, gold, silver, EC, glycerol, and chitosan, are approved as edible, i.e., safe to eat and not toxic upon ingestion, by European Food Safety Authority (EFSA), and the amount per transistor is well below the Allowed Daily Intake (ADI).^[^ ^97^, ^98^, ^99^, ^100^, ^101^ ^]^

However, the gold and silver nanoparticle‐based inks used to fabricate our metallic contacts do not fall within the same EFSA/Food and Drug Administrations regulations and they will need further assessment of their cytotoxicity.

An estimate of the amount of each edible material used for each transistor is reported in **Table** **2**:

**Table 2:** Estimated amount of each material per single device and corresponding ADI as per EFSA.

Material	Dose per device	ADI	Reference
CuPc	80 ng	N.A. (Daily ingested amount ~ 1 mg)	This manuscript
EC	≈3 mg	660–900 mg kg^−1^ day^−1^ (E462)	[97]
Printed gold	4 µg	N.A. (1.32 µg kg^−1^ day^−1^ for E175 edible gold)	[98]
Printed silver	14 µg	N.A. (12 µg kg^−1^ day^−1^ for E174 edible silver)	[99]
Chitosan	0.2 mg	3 g day^−1^	[100]
Glycerol	0.04 mg	No need for ADI	[101]

## 6 Conclusion

With this work, we propose the use of a commercial pigment employed for cosmetics, CuPc, as a semiconductor for applications in edible electronics. CuPc is particularly noteworthy due to its low bioavailability and limited solubility in water, saliva, and gastric fluids (see Figure S10, Supporting Information), making it a highly promising choice for integration into safely ingestible devices intended for use within the human body. As the first goal, we focused on whether any adverse reaction in humans was documented during the ≈15‐year period in which CuPc‐based tooth‐whitening toothpastes have been commercially available, which revealed no reported cases. Second, we sought to estimate the quantity of CuPc ingested through the accidental swallowing of toothpaste, yielding an estimated daily intake of up to ≈1.5 mg. Alongside this, we have experimentally quantified the daily intake coming from the whitening layer left on top of the teeth surface, calculated at ≈0.5 mg per day for a widely commercially available toothpaste. Consequently, despite the current absence of regulatory approval for the use of CuPc in food‐related applications, the substantial body of clinical trials available in the literature, along with our teeth brushing simulation test, collectively offer compelling and unambiguous evidence supporting the safe ingestion of up to ≈2 mg per day of CuPc.

Next, the feasibility of efficient and fully edible CuPc‐based electronics was addressed, starting from the demonstration of fully edible OFETs. Through an effort of process parametrization, we have successfully developed fully edible EGOFETs with operational voltages < 1 V, solely relying on scalable deposition techniques, including thermal evaporation and inkjet printing.^[^ ^102^, ^103^, ^104^ ^]^ These transistors, comprising EC (E462), gold (E175), silver (E174), and chitosan, exhibit state‐of‐the‐art electronic performances, characterized by On/Off ratio up to 10^3^, minimal hysteresis, low leakage current, and prolonged shelf‐life stability. Specifically, they demonstrated stable functionality when stored in ambient air conditions for over one year. The small loss of performances of the fully edible transistors to reference OFETs points toward the need for an additional engineering effort for tuning the surface energy of the edible substrate, which could induce an even better growth of CuPc crystallites (see Figure S2, Supporting Information). Considering 80 ng per device,^[^ ^96^ ^]^ more than 10^4^ transistors can be fabricated by using < 1 mg of CuPc, which would be sufficient even to envision the realization of edible microprocessors for complex electronics applications for the food industry and the healthcare sector.^[^ ^105^ ^]^

## 7 Experimental Section

### Materials

The gold ink DryCure Au‐J, 1010B (10 cps, 10 wt.%) was purchased from C‐INK Co., Ltd, while AgNPs ink (Silverjet DGP‐40LT‐15C) was purchased from Sigma–Aldrich.

Copper(II) Phthalocyanine powder (99.9% purity), EC powder (48.0‐49.5% (w/w) ethoxyl basis), chitosan powder (low molecular weight), glycerol and acetic acid were purchased from Sigma Aldrich.

Poly(chloro‐p‐xylene)‐C (Parylene‐C) dimer was purchased from Specialty Coating Systems.

### Device Fabrication

The transistors with a TGBC configuration were fabricated on top of corning glass, ultrasonically cleaned in acetone, IPA, and DI water for 5 min each, and then cleaned in O_2_ plasma for 5 min.

A 450 nm‐thick layer of poly(chloro‐p‐xylene)‐C was deposited by CVD with a SCS Labcoater 2—PDS2010 system and Au electrodes with *L* = 20 µm and *W* = 27 mm were inkjet‐printed on top and then sintered on a hotplate at 120 °C for 15 min.

CuPc was thermally evaporated in a Moorfield minilab 026 LTE evaporator with a base pressure between 3 × 10^−7^ and 6 × 10^−7^ mTorr and with a rate of 0.05 Å s^−1^.

A second 450 nm‐thick parylene‐C layer was CVD‐deposited on top of the evaporated CuPc layer.

The Ag gate was inkjet‐printed with a Fujifilm Dimatix DMP‐2831 with a Samba cartridge and then sintered at 120 °C for 1 h.

For the edible devices with a TGBC configuration, the EC films used as substrates and the chitosan‐based electrolyte were prepared following the recipe by Lamanna et al. and Sharova et al.^[^ ^56^ ^,^ ^106^ ^]^ Au electrodes with *L* = 20 µm and *W* = 27 mm were inkjet‐printed onto the EC films after an O_2_ plasma for 60 s at 20%.

Chitosan solution was drop‐casted on top of the CuPc layer in correspondence to the active area in a volume of 3 µL and then dried on a hotplate at 80 °C for 15 min.

The Ag gate was then printed on top and sintered for 15 min at 100 °C on a hotplate.

### Characterization

The electrical measurements of the characteristics of the devices were performed in air using an Agilent B1500A Semiconductor Parameter Analyzer.

The UV–vis spectra were recorded using a double‐beam Perkin Elmer l1050 spectrophotometer in the 500–800 nm range.

The AFM images were acquired through an Agilent 5500 atomic force microscope operated in acoustic mode.

The XRD patterns of the samples were recorded using a Bruker D8 Advance equipped with a Cu Kα1 (λ = 1.544060 Å) anode, operating at 40 kV and 40 mA.

### Ingestion Quantification of CuPc

The study protocol was approved by the Ethical Committee of the Dental Clinic of Vojvodina, approved 15 February 2023, protocol no. 127/23. All teeth were collected with the informed consent of the patient and written consent signed by the parents or caregivers. Experimental teeth were placed in two pairs of total dental prostheses, and the brushing was performed in a non‐Arcon type semi‐adjustable articulator. A total of 28 teeth were included in this study, with four incisors, two canines, four premolars, and four molars per jaw. The teeth were extracted for orthodontic reasons, surgical reasons, or due to severe periodontitis and were either intact or had adequate restorations. Before the experiment, the teeth were stored in saline for no longer than 28 days. To prepare the teeth for the experiment, they were cleaned ultrasonically to remove debris and periodontal ligament. The crowns were then separated from the roots at the cemento‐enamel junction using a diamond disk with water cooling. The crowns were arranged in wax according to the principles of tooth arrangement and then replaced with an acrylic base. Two prostheses were fabricated and placed in an articulator for tooth brushing simulation.

The tooth brushing procedure was repeated four times over two days by the same participant using a soft brush (Colgate Slim Soft, Colgate).

For every tooth brushing cycle, 0.250 g of toothpaste Colgate Advanced White was used. The brush was also inclined to execute vertical brushstrokes to cleanse the interior areas of the frontal teeth. After brushing the dentures were rinsed with water for 5 s, and then stored in deionized water following the experiment. Following the tooth brushing sessions, the whole liquid was collected and analyzed. Quantification of Cu content was performed with an atomic absorption spectrometer (AAS, Varian AA240/GTA 120) with deuterium background correction. The limit of detection for Cu was 0.0785 mg kg^−1^.

## Conflict of Interest

The authors declare no conflict of interest.

## Supporting information

Supporting Information

## Data Availability

Research data are not shared.
