# Supplementary material for: A Fully Edible Transistor Based on a Toothpaste Pigment
Source: Adv Sci (Weinh). 2024 Sep 16;11(41):2404658. doi: 10.1002/advs.202404658 (PMC11538660; doi:10.1002/advs.202404658)
Supplement: Supplementary file 1 — Supporting Information [file ADVS-11-2404658-s001.pdf]

ADVANCED SCIENCE Open Access

Supporting Information

for *Adv. Sci.*, DOI 10.1002/advs.202404658

A Fully Edible Transistor Based on a Toothpaste Pigment

Elena Feltri, Pierluigi Mondelli, Bojan Petrović, Fabrizio Mario Ferrarese, Alina Sharova, Goran Stojanović, Alessandro Luzio and Mario Caironi**

Supporting Information

A Fully Edible Transistor Based on a Toothpaste Pigment

Elena Feltri, Pierluigi Mondelli, Bojan Petrović, Fabrizio Mario Ferrarese, Alina Sharova, Goran Stojanović, Alessandro Luzio and Mario Caironi**

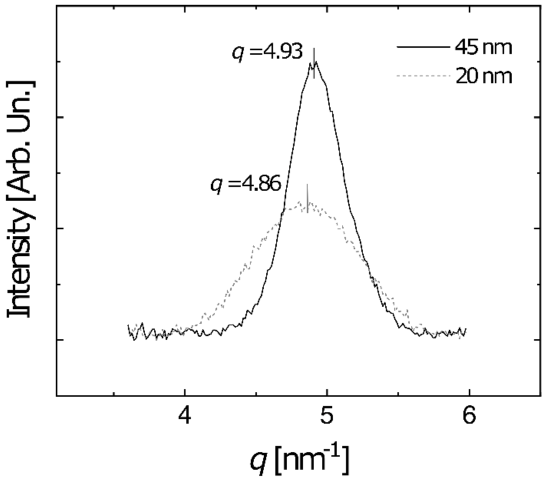

Figure S1: XRD diffractograms on 20 and 45 nm-thick CuPc films evaporated on glass substrates.

XRD performed at different CuPc thicknesses shows the α-phase main reflection (2 0 0), centered at q = 4.90 nm^{-1}, in agreement with measurements performed on thicker films (Figure 2a).

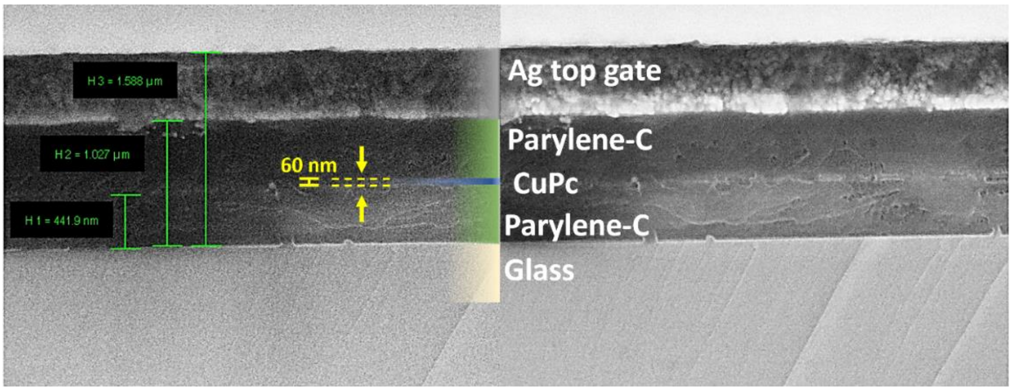

Figure S2: SEM cross-sectional image of a 60 nm-thick CuPc solid-state transistor.

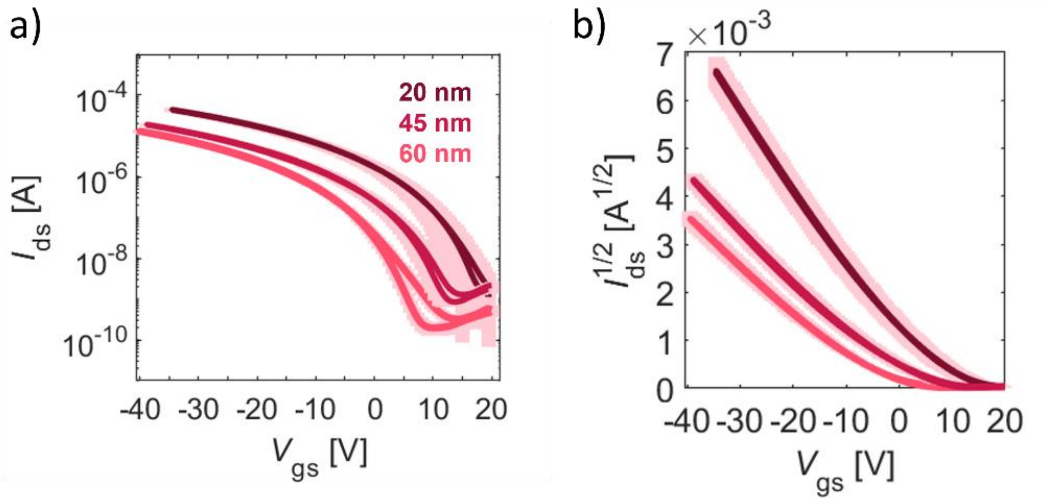

Figure S3: *a)* I_{ds} vs V_{gs} curve plotted in logarithmic scale and *b)* $I_{ds}^{1/2}$ vs V_{gs} curves plotted in linear scale up to the maximum V_{gs} value for which the devices are still in saturation regime, taking into account the threshold voltage shift towards positive potentials while scaling down the CuPc thickness (V_{th} = 5.2 V, 0.8 V, and -3.4 V respectively for the 20 nm, 45 nm, and 60 nm films).

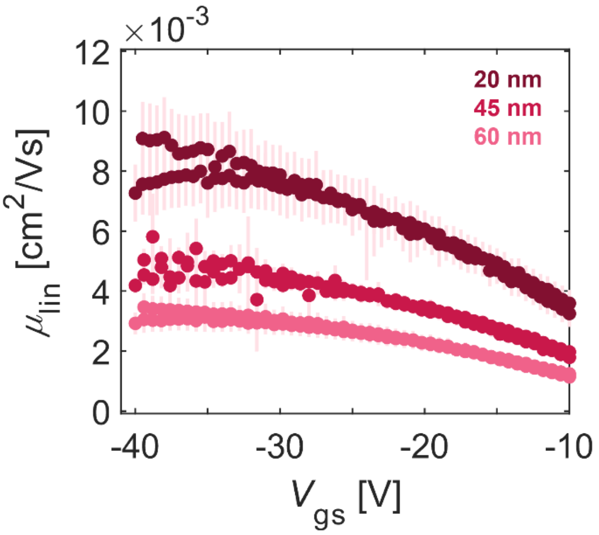

Figure S4: Field-effect mobility curves calculated in linear regime at V_{ds} = -5V of the reference non-edible TGBC devices for the three different CuPc film thicknesses, 20, 45, and 60 nm.

The calculated field-effect mobility values in the linear regime, V_{ds} = -5V, confirm the quasi-ideality of the devices since they correspond to those calculated in the saturation regime (V_{ds} = -40V), shown in Figure 3c and reported in Table 1.

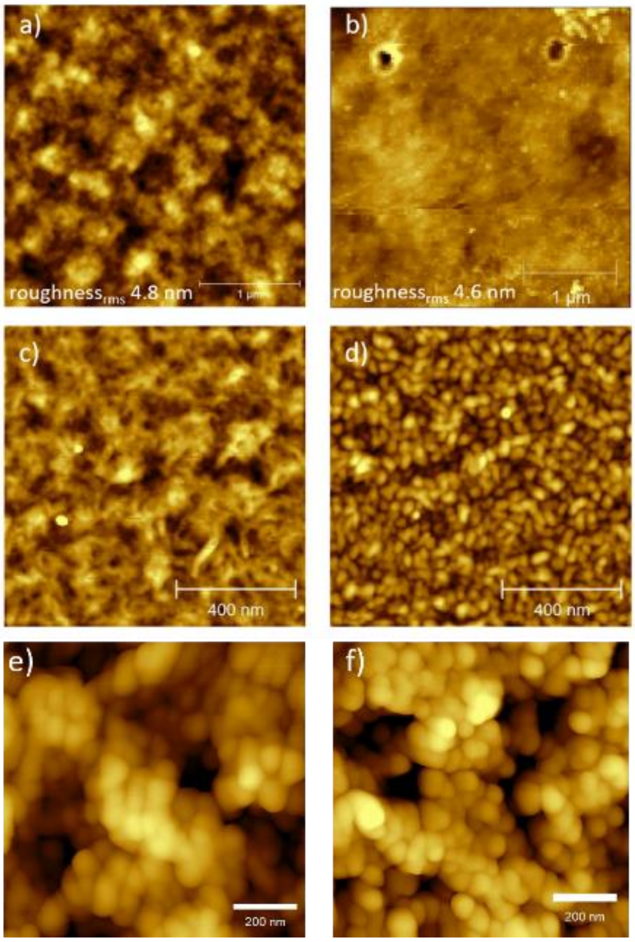

Figure S5: AFM topography images of *a)* parylene-C, *b)* ethyl cellulose, *c)* 20 nm thick evaporated CuPc on top of parylene-C, *d)* 20 nm thick CuPc on top of ethyl cellulose, *e)* 20 nm thick CuPc on top of printed gold contacts, *f)* printed gold contacts, reproduced from Ref. 56 with permission from the Royal Society of Chemistry.

The different AFM images show that CuPc crystallites tend to grow in a more elongated fashion on top of parylene-C, giving instead rise to smaller grains onto ethyl cellulose. This difference, possibly induced by the different surface energies of the two substrates, could be the reason behind the small loss in performances of EGOFETs growth onto ethyl cellulose. The CuPc morphology on the printed gold electrodes shows conformity with the nanostructured gold layer.

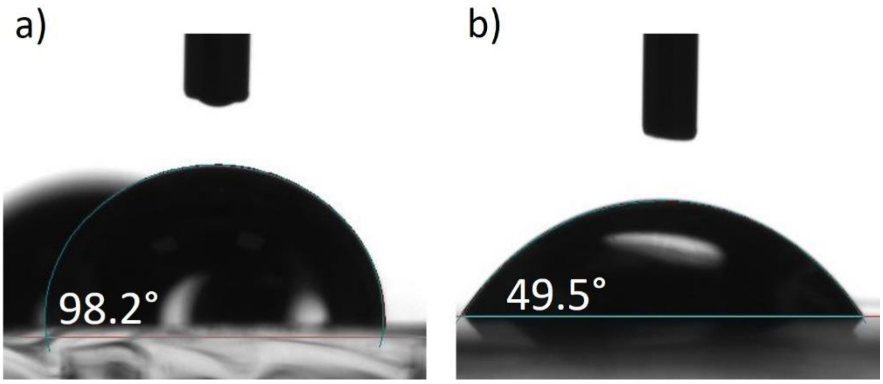

Figure S6: Contact angle measurements on *a)* parylene-C and *b)* ethyl cellulose.

The contact angle measurements show the different hydrophobic/hydrophilic nature of the two materials, which in turn influences the CuPc crystallite growth.

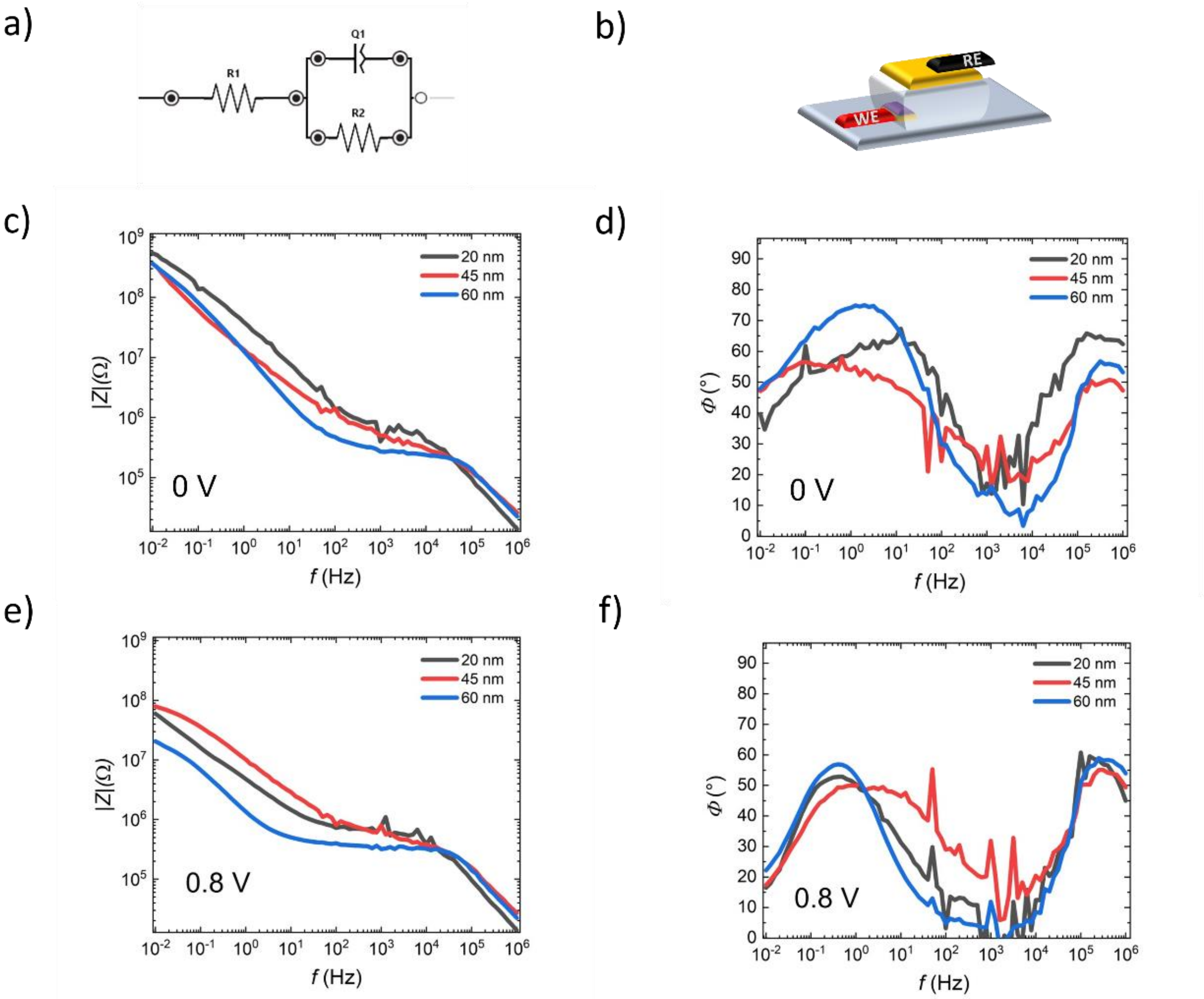

Figure S7: *a)* Randler's equivalent circuit of the Electrochemical Impedance Spectroscopy (EIS) measurements. *b)* Sketch of the metal-electrolyte-metal architecture used to characterize the properties of the CuPc-chitosan interface, with gold for both Working Electrode (WE) and Reference Electrode (RE). *c)* Bode plot of the modulus of the impedance Z(Ω) as a function of the frequency in a range of 10 mHz-1 MHz at 0 V. *d)* Bode plot of the phase φ(°) of the impedance Z(Ω) as a function of the frequency in a range of 10 mHz – 1 MHz at 0 V. *e)* Bode plot of the modulus of the impedance Z(Ω) as a function of the frequency in a range of 10 mHz-1 MHz at 0.8 V. *f)* Bode plot of the phase φ(°) of the impedance Z(Ω) as a function of the frequency in a range of 10 mHz – 1 MHz at 0.8 V.

Electrochemical impedance spectroscopy (EIS) was performed on a metal-electrolyte-metal architecture to characterize the properties of the CuPc-chitosan interface.
EIS analysis was performed in the range of 10 mHz - 1 MHz, applying a sine wave (V_{peak} = 100 mV) and a DC bias between 0 mV and 800 mV. Figure S7a shows the Randler circuit used to model an Electrolyte-Metal interface system, where R1 is the resistance of the electrolyte, R2 is the resistance due to charge transfer phenomena, and Q1 represents non-ideal capacities such as EDL. Using the Hsu-Mansfeld equation, the effective EDL capacity was then extracted from the fitting with the equivalent circuit. Figure S7b shows the experimental setup used to perform the EIS measurements.
Figures S7c and S7d show the curves of the absolute value of the impedance Z(Ω) and its phase φ(°) as a function of the frequency in the conditions of DC bias 0 mV for the three different CuPc thicknesses.
Figures S7e and S7f show the curves of the absolute value of the impedance Z(Ω) and its phase φ(°) as a function of the frequency in the conditions of DC bias 800mV for the three different CuPc thicknesses.

Sample	C 0 V (μF/cm^2)	C 0.4 V (μF/cm^2)	C 0.6 V (μF/cm^2)	C 0.8 V (μF/cm^2)
CuPc 20 nm	1.3 ± 0.2	2.4 ± 1.4	3.4 ± 2.6	4 ± 2.3
CuPc 45 nm	1.6 ± 0.8	0.9 ± 0.1	1.4 ± 0.9	1.8 ± 1.1
CuPc 60 nm	0.9 ± 0.3	1.9 ± 0.6	3.6 ± 1.6	6.5 ± 2.8

Table S1: EDL capacities experimental values extracted at different DC biases for the three different CuPc film thicknesses (20, 45, and 60 nm).

The extracted values in Table S1 agree with the theoretical values of an EGOFET.[90][91]

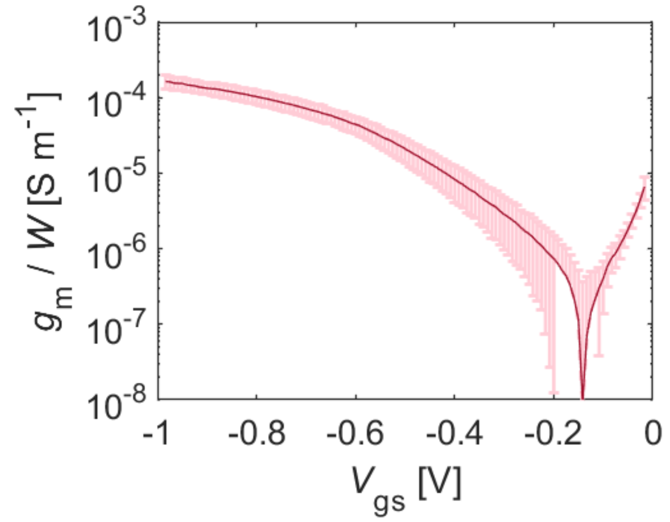

Figure S8: Transconductance normalized to the channel width of the fully edible devices.

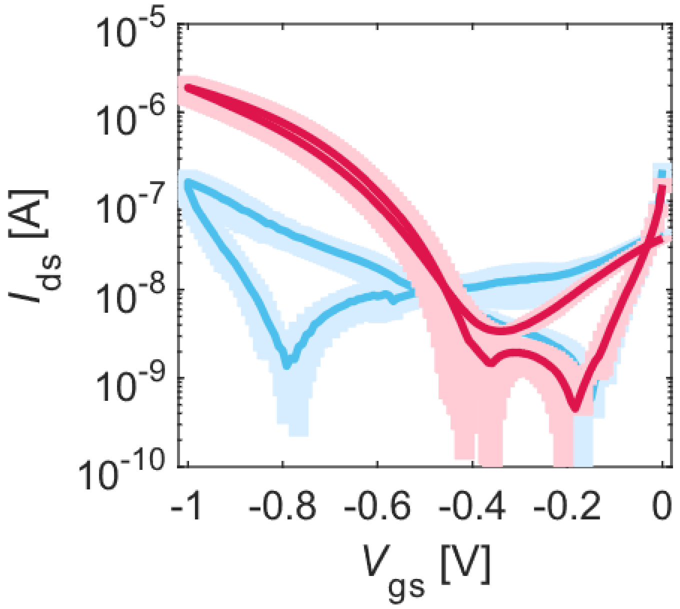

Figure S9: Transfer characteristics of chitosan-gated EGOFET on parylene-C as substrate, with V_{ds} = -500 mV, averaged over 5 devices for 20 nm-thick CuPc films.

The transfer characteristics of chitosan-gated EGOFET with parylene-C as a substrate yield a mean current of 2 μA, which is double the value of chitosan-gated EGOFET onto ethyl cellulose.

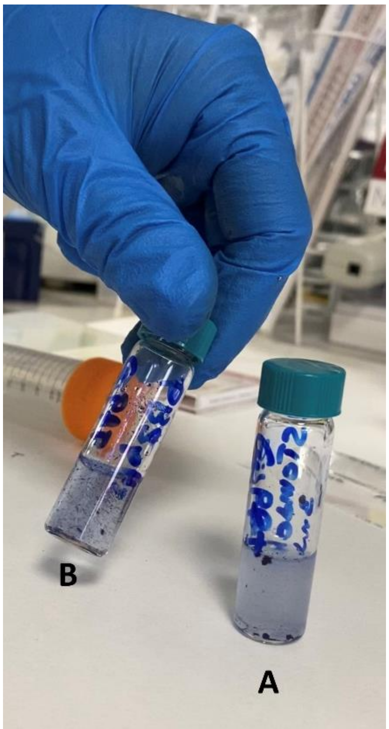

Figure S10: CuPc digested in saliva and stomach environments (2 h of digestion) (A) and in simplified saline buffer at stomach pH (B)**.**

CuPc powder was digested by the cascade in vitro dissolution assay following the method presented in Di Cristo et al. 2014.[1-4] Furthermore, an additional sample was tested by incubating the powder only in the presence of buffer at pH 2.0 simulating the gastric juice. In both environments, it is evident a great aggregation tendency of the powder due to its complete insolubility at these conditions. The presented behavior may be indicative of the biotransformation of CuPc in the OGI tract, namely, it will greatly aggregate to be cleared by the feces. This observation found support in present literature data that report the absence of dissolution of copper-based nano pigments in other human-relevant environments e.g. the lung, for which the physical clearance from the lung is accompanied by aggregation and/or reprecipitation.

References

[1] J. Koltermann-Jülly, J. G. Keller, A. Vennemann, K. Werle, P. Müller, L. Ma-Hock, R. Landsiedel, M. Wiemann, W. Wohlleben, *NanoImpact* **2019**, *14*, 100154.

[2] L. Di Cristo, V. C. Ude, G. Tsiliki, G. Tatulli, A. Romaldini, F. Murphy, W. Wohlleben, A. G. Oomen, P. P. Pompa, J. Arts, V. Stone, S. Sabella, *Part Fibre Toxicol* **2022**, *19*, 68.
[3] P. Bove, M. A. Malvindi, S. S. Kote, R. Bertorelli, M. Summa, S. Sabella, *Nanoscale* **2017**, *9*, 6315.
[4] I. S. Sohal, Y. K. Cho, K. S. O'Fallon, P. Gaines, P. Demokritou, D. Bello, *ACS Nano* **2018**, *12*, 8115.
